# Comparison of metastasis between early-onset and late-onset gastric signet ring cell carcinoma

**DOI:** 10.1186/s12876-020-01529-z

**Published:** 2020-11-14

**Authors:** Qiang-Ping Zhou, Yong-Hua Ge, Cheng-Yuan Liu

**Affiliations:** 1 Department of Emergency Surgery, The First Affiliated Hospital of Nanchang University, 17 Yongwaizheng Street, Nanchang, 330006 Jiangxi China; 2 Department of Prevention and Health Care, Jiangxi Cancer Hospital, Nanchang, China

**Keywords:** Early-onset gastric cancer, Signet ring cell carcinoma, Distant metastasis, SEER

## Abstract

**Background:**

There is little knowledge to date about the distant metastasis of early-onset gastric signet ring cell carcinoma (SRCC) or the difference in metastasis based on age. Therefore, we conducted a comprehensive retrospective study using the Surveillance, Epidemiology, and End Results (SEER) database and data from our hospital.

**Methods:**

Patients were collected from the SEER database and our hospital. Univariate and multivariate logistic regression analyses and propensity score matching (PSM) were used to identify risk factors for metastasis. K-M survival curves were generated to analyse patient survival.

**Results:**

In total, we retrieved 2052 EOGC patients diagnosed with SRCC from the SEER database and included 403 patients from our hospital. K-M survival curves showed that late-onset SRCC patients had worse survival than early-onset patients but that late-onset SRCC patients were less likely to have distant metastasis, as validated by SEER data (OR = 0.462, 95%CI, 0.272–0.787; P = 0.004) and our data (OR = 0.301, 95%CI, 0.135–0.672; P = 0.003). Multivariate logistic regression and PSM analysis revealed that age of 45 or younger was an independent risk factor for distant metastasis.

**Conclusion:**

Our study showed that distant metastasis was more common in early-onset SRCC than in late-onset SRCC. However, further studies are needed to explore the potential aetiologic basis for this disparity.

## Background

Gastric cancer (GC) is one of the most common tumours among all kinds of malignant carcinomas worldwide, with GC-associated death ranking fourth [1]. Currently, there are several classification systems for GC, including the Lauren classification and WHO classification. The Lauren classification divides GC into intestinal and diffuse types according to histological subtype; the WHO classification describes GC as four types, including signet ring cell carcinoma (SRCC) and mucinous types [2, 3]. SRCC is defined as a histological type GC in which tumour cells are composed of many mucins (> 50% size of cell) and the nucleus is squeezed into the ridge of cell. SRCC belongs to diffuse-type GC and undifferentiated GC, predicting poorer prognosis compared to other types of GC [4]. Regarding the survival of SRCC, the 5-year disease-free survival (DFS) rate is 86.9% for stage I patients, 38.3% for stage II patients, and only 16.2% for stage III patients [5]. Compared to other types of GC, SRCC is considered an unfavourable predictor of prognosis; additionally, SRCC serves as an independent risk factor for hepatic metastasis and peritoneal metastasis [4]. In addition, according to age at diagnosis, GC is divided into early-onset GC (EOGC) and late-onset GC (LOGC), of which the former is defined as a tumour diagnosed before 45 years of age [6]. With regard to the trend of incidence in GC, the incidence of diffuse-type GC is increasing, and that of intestinal-type GC is declining; the incidence of EOGC has also been steadily increasing, and that of LOGC is decreasing [6, 7]. Compared to LOGC, EOGC is quite different as a worrisome malignant tumour because of less exposure to environmental factors [8].

Given the disparities between EOGC and LOGC and the high malignancy of SRCC, we sought to investigate the rate of metastasis for early- and late-onset SRCC. Through this study, we aim to enhance our clinical understanding of these cancer types and provide evidence for therapeutic selection, with the ultimate goal of improved outcomes. In our study, we collected 2052 SRCC patients from the SEER database, including 234 EOGC and 1818 LOGC patients who were diagnosed from 2010 to 2015, and performed a comprehensive analysis that was validated by an external group consisting of 403 SRCC patients, including 54 EOGC and 349 LOGC patients.

## Methods

### Patients

**Fig. 1:**
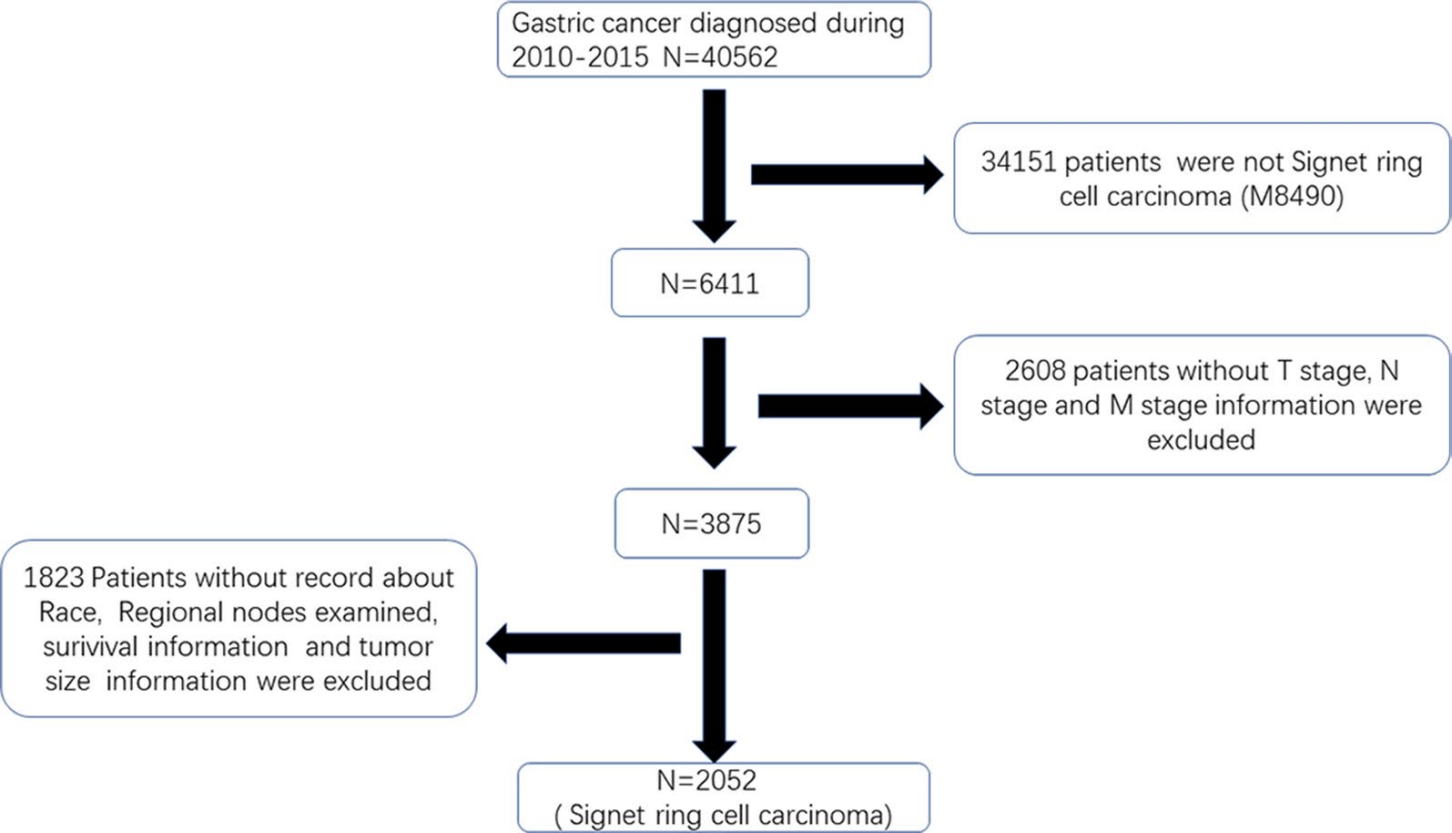
The flowchart of extracting patient information from the SEER database in our study

**Fig. 2:**
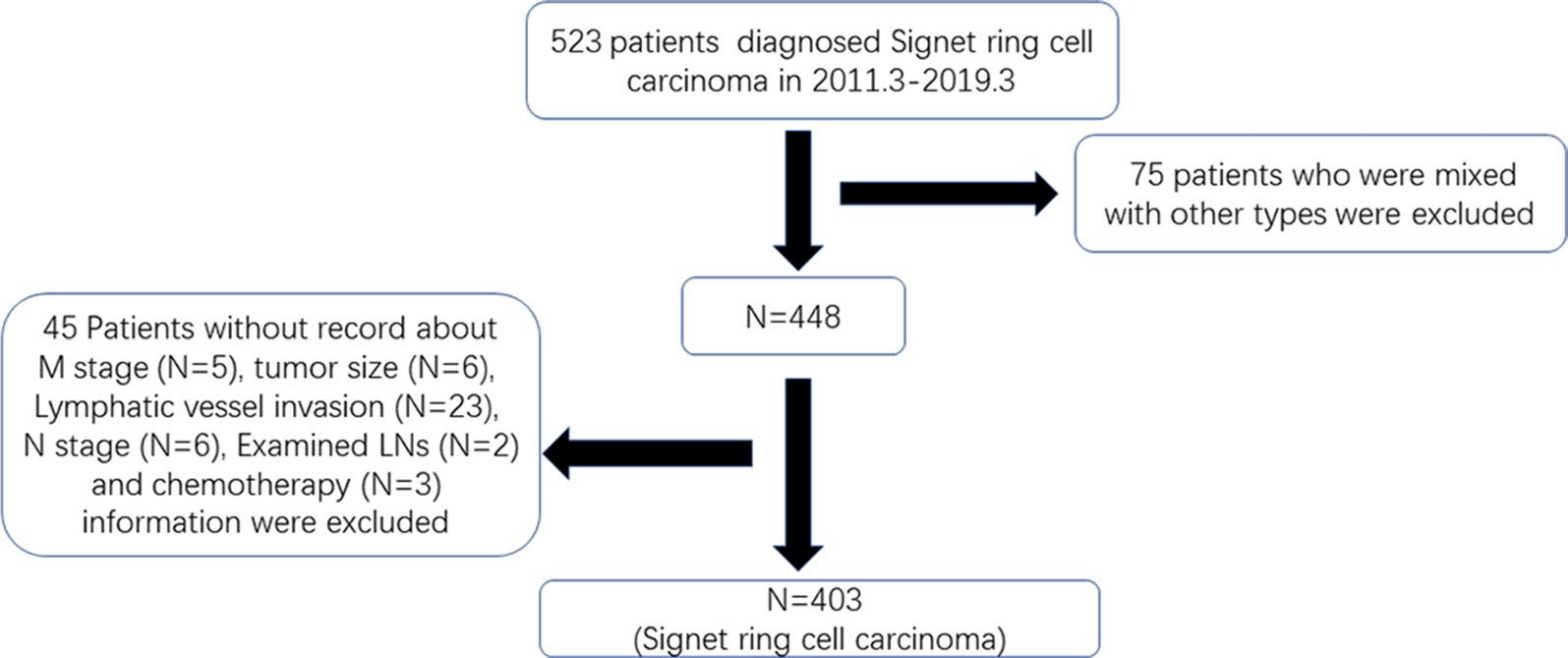
The flowchart of extracting patient information from the First Affiliated Hospital of Nanchang University in our study

All patients with GC in the SEER database were retrieved using National Cancer Institute’s SEER*Stat software (version 8.3.6). The patients did not give informed consent because the SEER database is free for public use. All patients underwent surgery. According to the International Classification of Diseases in Oncology (ICD-O-3), tumours with codes 8490 are identified as SRCC. In our study, patients were included according to the following criteria: (1) older than 20 who were diagnosed with GC by positive histology from 2010 through 2015; (2) SRCC histopathology; (3) survival information; and (4) detailed information, including age, race, grade, examined LNs, tumour size, T stage, N stage and M stage. Detailed information on the excluded patients is listed in Fig. 1. In addition, we extracted 403 patients diagnosed with SRC from March 2011 to March 2019 in the First Affiliated Hospital of Nanchang University. Patients were included according to the following criteria: (1) aged more than 20 years and underwent surgery, (2) diagnosed with SRCC by histology from March 2013 to March 2019, and (3) no serious chronic diseases, such as chronic renal failure. Patients were excluded according to the following criteria: (1) no record of TNM staging, tumour size, lymphatic vessel invasion or examined lymph nodes (LNs) or (2) chemotherapy before surgery. The study was approved by the Ethics Committee of the First Affiliated Hospital of Nanchang University. The detailed information is shown in Fig. 2.

### Clinicopathological factors

The cases in the SEER database and from our hospital were divided into the EOGC group and LOGC group based on clinicopathological variables. The patients from the SEER database and our hospital were divided into two age groups: < = 45 and > 45 years. Race was classified into three types: white, black and other. T stage was recorded as T1, T2, T3 and T4. LNM was described as N0 (negative), N1 (1–2 positive LNs), N2 (3–6 positive LNs) and N3 (> 6 positive LNs). M1 (Yes) indicates a positive M stage. Tumour size was categorized into 4 groups: ≤ 2 cm, ≤ 5 cm, and > 5 cm. With respect to examined LNs, the cut-off value was 16 according to previous studies [9]. Primary sites were recorded as cardia, fundus, body, antrum, and overlapping lesion/NOS. The status of chemotherapy was recorded as no or yes. The medicines used for chemotherapy included XELODA, tegafur and oxaliplatin. The different methods and courses of chemotherapy were determined according to the TNM stage of the patients [10]. All information of patients from the SEER database is shown in Table 1; that of patients from our hospital is shown in Table 2. The primary observation indicator was distant metastasis. The status of distant metastasis was diagnosed when the patients were first admitted to the hospital.

**Table 1:** Basic information of extracted patients from SEER diagnosed in 2010–2015

Variables	Total	Early-onset SRCC	Late-onset SRCC	P value
Total	2052	234	1818	
*Sex*				0.0005
Male	1034 (50.39%)	94 (40.17%)	949 (52.2%)	
Female	1009 (49.61%)	140 (59.83%)	869 (47.8%)	
*Race*				0.739
White	1381 (67.3%)	157 (67.1%)	1224 (67.33%)	
Black	252 (12.28%)	32 (13.67%)	220 (12.1%)	
Other	419 (20.42%)	45 (19.23%)	374 (20.57%	
*Primary site*				0.013
Cardia	283 (13.79%)	20 (8.55%)	263 (14.47%)	
Fundus	54 (2.63%)	7 (2.99%)	47 (2.59%)	
Antrum	1076 (52.44%)	118 (50.43%)	958 (52.7%)	
Body	232 (11.31%)	39 (16.67%)	193 (10.6%)	
Overlappping/NOS	407 (18.83%)	50 (21.37%)	357 (19.64%)	
*T stage*				0.657
Tis/T1	408 (19.88%)	45 (19.23%)	363 (19.97%)	
T2	224 (10.92%)	30 (12.82%)	194 (10.67%)	
T3	694 (33.82%)	73 (31.2%)	621 (34.16%)	
T4	726 (35.38%)	86 (36.75%)	640 (35.2%)	
*N stage*				0.738
N0	731 (35.62%)	86 (36.75%)	645 (35.48%)	
N1	357 (17.4%)	37 (15.81%)	320 (17.6%)	
N2	346 (16.86%)	44 (18.8%)	302 (16.61%)	
N3	638 (31.09%)	67 (28.63%)	551 (30.3%)	
*M stage*				0.000
M0	1818 (88.6%	188 (80.34%)	1630 (89.66%)	
M1	234 (11.4%)	46 (19.66%)	188 (10.34%)	
*Tumor number*				0.000
1	1629 (79.39%)	217 (92.74%)	1412 (77.67%)	
> 1	423 (20.61%)	17 (7.26%)	406 (22.33%)	
*Tumor size*				0.99
≤ 2 cm	430 (20.96%)	51 (21.79%)	389 (21.4%)	
≤ 5 cm	793 (38.65%)	90 (38.46%)	703 (38.67%)	
> 5 cm	819 (39.91%0	93 (39.74%)	726 (39.93%)	
*Examined LNs*				0.227
≤ 16	997 (48.59%)	105 (44.87%)	892 (49.06%)	
> 16	1055 (51.41%)	129 (55.13%)	926 (50.94%)	
*Chemotherapy*				0.000
No	1295 (63.11%)	90 (38.46%)	1205 (66.28%)	
Yes	757 (36.89%)	144 (61.54%)	613 (33.72%)

**Table 2:** Basic information of included patients from our hospital diagnosed in 2003–2019

Variables	Total	Early-onset GSRCC	Late-onset GSRCC	P value
Total	403	54	349	
*Sex*				0.000
Male	259 (64.27%)	22 (40.74%)	237 (67.91%)	
Female	144 (35.73%)	32 (59.25%)	112 (32.09%)	
*Hypertension*				0.121
No	353 (87.59%)	51 (94.44%)	302 (86.53%)	
Yes	50 (12.41%)	3 (5.56%)	47 (13.47%)	
*Diabetes*				0.028
No	363 (90.07%)	53 (98.15%)	310 (88.82%)	
Yes	40 (9.93%)	1 (1.85%)	39 (11.17%)	
*Smoke*				0.079
Never	288 (71.46%)	44 (81.48%)	244 (69.91%)	
Yes	115 (28.54%)	10 (18.51%)	105 (30.09%)	
*Primary site*				0.237
Cardia	9 (2.23%)	1 (1.85%)	8 (2.29%)	
Fundus	8 (1.99%)	1 (1.85%)	7 (2%)	
Antrum	251 (62.28%)	29 (53.7%)	222 (63.61%)	
Body	99 (24.57%)	20 (37.04%)	79 (22.64%)	
Overlappping/NOS	36 (8.93%0	3 (5.56%)	33 (9.46%)	
*T stage*				0.79
T1	69 (17.12%)	10 (18.51%)	59 (16.91%)	
T2	55 (13.65%)	5 (9.26%)	50 (14.33%)	
T3	52 (12.9%)	7 (12.96%)	45 (12.89%)	
T4	227 (56.33%)	32 (59.25%)	195 (55.87%)	
*N stage*				0.997
N0	139 (34.49%)	18 (33.33%)	121 (34.67%)	
N1	60 (14.89%)	8 (14.81%)	52 (14.89%)	
N2	93 (23.08%)	13 (24.07%)	80 (22.92%)	
N3	111 (27.54%)	15 (27.78%)	96 (27.51%)	
*M stage*				0.036
M0	360 (89.33%)	43 (79.63%)	317 (90.83%)	
M1	43 (10.67%)	11 (20.37%)	32 (9.17%)	
*Lesion classification*				0.064
Infiltration type	20 (4.96%)	7 (12.96%)	17 (4.87%)	
Ulcerative type	53 (13.15%)	40 (74.07%)	286 (81.95%)	
Protuberant type	326 (80.89%)	7 (12.96%)	46 (13.18%)	
*Lymphatic vessel invasion*				0.023
No	185 (45.91%)	17 (31.48%)	168 (48.14%)	
Yes	218 (54.09%)	37 (68.52%)	181(51.86%)	
*Tumor size*				0.562
≤ 2 cm	77 (19.11%)	12 (15.22%)	65 (18.62%)	
≤ 5 cm	150 (37.22%)	22 (40.74%)	128 (36.68%)	
> 5 cm	176 (43.67%)	20 (37.04)	156 (44.7%)	
*Examined LNs*				0.924
≤ 16	58 (14.39%)	8 (14.81%)	50 (14.33%)	
> 16	345 (85.61%)	46 (85.19%)	299 (85.67%)	
*Chemotherapy*				0.017
No	273 (67.74%)	29 (53.7%)	244 (69.91%)	
Yes	130 (32.26%)	25 (46.3%)	105 (30.09%)	
*Methods of surgery*				0.383
Conditional surgery	33 (8.19%)	5 (9.26%)	28 (8.02%)	
Laparoscopic surgery	346 (85.86%)	48 (88.89%)	298 (85.39%)	
Robotic surgery	24 (5.96%)	1 (1.85%)	23 (6.59%)	

### Statistical analysis

For basic statistics, patients were divided into two groups, namely, LOGC and EOGC, and Pearson’s chi-squared test was utilized to investigate the association among categorical variables. To explore potential risk factors for distant metastasis, we performed univariate and multivariate Cox regression analyses, and the results are reported using the odds ratio (OR) with the 95% confidence interval (CI). K-M survival curve analysis was performed for the OS and CSS of patients with SRC.

Regarding the imbalance between LOGC and EOGC groups, we performed propensity-score matching (PSM) to obtain new data for analysis with the MatchIt package in R software. The value of the calipre was set as 0.02, and the effect was evaluated based on the P value. The detailed process was as follows. First, we calculated the propensity scores of each patient according to age (LOGC and EOGC) with the multivariate logistic regression model. Then, we matched patients between the two groups at a ratio of 1:1. Next, we analysed differences in all variables between the EOGC and LOGC groups with the chi-squared test. Finally, we explored the correlation between age and distant metastasis using a univariate logistic regression model.

All statistical analyses were performed with R software (version 3.6.1, StataCorp LLC, College Station, Texas). The chi-square test for the categorical variable, Student’s t-test for continuous variables with Gaussian distribution, and the nonparametric Kruskal–Wallis rank sum test for continuous variables with nonnormally distributed data or ordinal categorical variables were used for comparisons among different patient groups. The chi-squared test was carried out with SPSS (version 24.0). The results were considered to be statistically significant when the P value was less than 0.05.

## Results

### Basic information of extracted cases

According to the inclusion criteria and exclusion criteria, we collected patients diagnosed with SRC from the SEER database and included patients with SRC histology from our hospital. As shown in Figs. 1 and 2, we extracted 2052 cases from the SEER database and 403 from our hospital. The basic information of the patients in the two groups is listed in Tables 1 and 2. For patients from the SEER database, patients diagnosed with early-onset SRCC were more frequently female (59.83% vs 47.8%, P < 0.001) and the tumour was located in the body of the stomach (16.7% vs 10.6%, P < 0.05) compared to late-onset SRCC. In addition, early-onset SRCC patients more often had distant metastasis (19.66% vs 10.34%, P < 0.001), though they more rarely had more than 2 tumours (7.26% vs 22.33%, P < 0.001). With regard to the basic information of patients from the First Affiliated Hospital of Nanchang University (Table 2), similarly, we found that early-onset SRCC patients tended to be female compared to late-onset SRCC patients (59.25% vs 32.09%, P < 0.05). In addition, the proportion of patients with metastatic and lymphatic invasion in early-onset SRCC was larger than that in late-onset SRCC (P < 0.05). Interestingly, we found that the proportion of those undergoing chemotherapy for early-onset SRCC was obviously higher than that for late-onset SRCC (46.3% vs 30.09%, P = 0.017).

### Survival analysis and identification of risk factors for metastasis

**Fig. 3:**
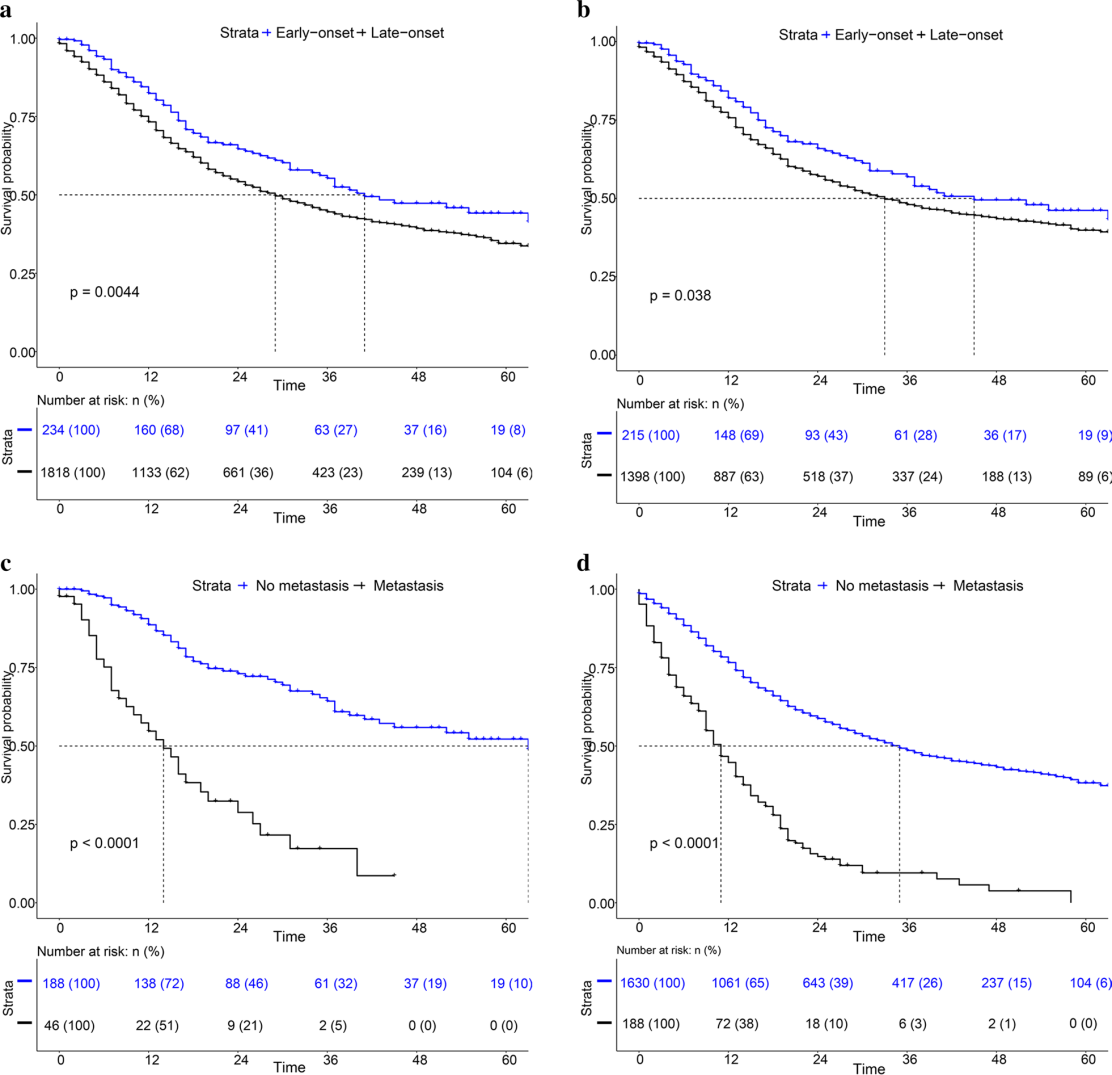
Survival of SRCC patients from the SEER database with early onset and late onset. **a** Overall survival of early-onset SRCC and late-onset SRCC patients. **b** Cancer-specific survival of early-onset SRCC and late-onset SRCC patients. **c** Survival of early-onset SRCC with negative metastasis or positive metastasis. **d** Survival of late-onset SRCC with negative metastasis or positive metastasis

To investigate the survival of patients with early-onset and late-onset SRCC, we generated a K-M survival curve (Fig. 3). Regarding overall survival, patients with early-onset SRCC had a 1-year survival rate of 74.25%, a 3-year survival rate of 56.32% and a 5-year survival rate of 45.84%; patients diagnosed with late-onset SRCC had a 1-year survival rate of 65.48%, a 3-year survival rate of 47.29% and a 5-year survival rate of 36.45%, with significant differences (Fig. 3a, P = 0.0044). Similarly, for cancer-specific survival, early-onset SRCC patients had a better survival than those who had late-onset SRCC (Fig. 3b, P = 0.038). In addition, we divided patients into negative metastasis and positive metastasis groups and drew K-M survival curves. For both early-onset SRCC and late-onset SRCC, patients with distant metastasis had obviously poorer survival than those with no metastasis (Fig. 3c, d). To identify potential risk factors for distant metastasis, we performed univariate and multivariate logistic regression analyses. For patients from the SEER database, we found that black ethnicity was a favourable factor compared to other races (OR = 0.462, 95%CI, 0.272–0.787; P = 0.004). Additionally, advanced T stage was an independent risk factor for distant metastasis. Interestingly, we found that age was an independent factor for metastasis and that patients diagnosed with early-onset SRCC more frequently developed metastasis (Table 3). To validate these findings, we analysed data from our own hospital and found that patients aged more than 45 years had metastasis less often than patients aged < = 45 years old (OR = 0.301, 95%CI, 0.135–0.672; P = 0.003) (Table 4). Moreover, smoking, advanced T stage and lymphatic vessel invasion were independent risk factors for metastasis (P < 0.05) (Table 4). Regarding confounding factors, we performed PSM using the two sets of data. As shown in Tables 5 and 6, we adjusted the imbalanced data (P > 0.05) and observed that early-onset SRCC patients were more likely to develop metastasis (P < 0.05).

**Table 3:** Univariate and Multivariate logistic regression analysis of EOGC and LOGC patients from SEER for metastasis

Variables	Univariate analysis	P value	Multivariate analysis	P value
*Sex*				
Male	Reference	–	Reference	–
Female	1.407(1.068–1.854)	0.015	1.242(0.925–1.668)	0.149
Age		0.000		
≤ 45	Reference	–	Reference	–
> 45	0.512(0.356–0.737)	0.000	0.492(0.333–0.725)	0.000
Race				
White	Reference	–	Reference	–
Black	0.512(0.305–0859)	0.011	0.462(0.272–0.787)	0.004
Other	0.809(0.568–1.153)	0.241	0.838(0.578–1.215)	0.351
*Primary site*				
Cardia	Reference	–		
Fundus	1.13(0.314–4.075)	0.852		
Antrum	2.269(1.282–4.015)	0.005		
Body	2.365(1.193–4.689)	0.014		
Overlappping/NOS	1.341(0.699–1.553)	0.054		
*T stage*				
Tis/T1	Reference	–	Reference	–
T2	3.766(1.271–11.16)	0.000	3.221(1.057–9.821)	0.04
T3	7.767(3.095–19.49)	0.000	5.686(2.114–15.298)	0.001
T4	21.879(8.899–53.79)	0.000	12.992(4.856–34.754)	0.000
*N stage*				
N0	Reference	–	Reference	–
N1	1.357(0.814–2.262)	0.242	0.792(0.463–1.356)	0.395
N2	2.517(1.606–3.943)	0.000	1.242 (0.766–2.012)	0.379
N3	4.206(2.889–6.123)	0.000	1.543(1.003–2.373)	0.048
*Tumor number*				
1	Reference	–		
> 1	0.84(0.59–1.195)	0.333		
*Tumor size*				
≤ 2 cm	Reference	–	Reference	–
≤ 5 cm	2.727(1.568–4.744)	0.000	1.273(0.692–2.34)	0.437
> 5 cm	5.511(3.24–9.373)	0.000	1.601(0.869–2.95)	0.131
*Examined LNs*				
≤ 16	Reference	–		
> 16	0.859(0.653–1.13)	0.278

**Table 4:** Univariate and Multivariate logistic regression analysis of EOGC and LOGC patients from our hospital for metastasis

Variables	Univariate analysis	P value	Multivariate analysis	P value
*Sex*				
Male	Reference	–		
Female	0.846(0.425–1.686)	0.635		
*Age*				
≤ 45	Reference	–	Reference	–
> 45	0.395(0.185–0.84)	0.016	0.301(0.135–0.672)	0.003
*Hypertension*				
No	Reference	–		
Yes	1.165(0.465–2.918)	0.745		
*Diabetes*				
No	Reference	–		
Yes	1.506(0.594–3.819)	0.389		
*Smoke*				
Never	Reference	–	Reference	–
Yes	2.177(1.142–4.152)	0.018	2.768(1.355–5.657)	0.005
*Primary site*				
Cardia	Reference	–		
Fundus	–	–		
Antrum	0.807(0.097–6.741)	0.843		
Body	1.412(0.164–12.121)	0.753		
*Overlappping/NOS*				
*T stage*				
T1	Reference	–	Reference	–
T2	4.102(0.794–21.193)	0.092	3.377(0.584–19.536)	0.174
T3	5.211(1.035–26.233)	0.045	2.141(0.312–14.684)	0.438
T4	4.714(1.094–20.317)	0.038	1.873(1.327–12.112)	0.047
*N stage*				
N0	Reference	–	Reference	–
N1	1.819(0.603–5.493)	0.288	1.028(0.306–3.458)	0.964
N2	2.719(0.651–4.726)	0.266	1.085(0.361–3.262)	0.884
N3	6.564(1.519–8.526)	0.004	2.102(1.064–5.858)	0.035
*Lesion classification*				
Infiltration type	Reference	–		
Ulcerative type	0.601(0.194–1.86)	0.377		
Protuberant type	0.408(0.093–1.794)	0.235		
*Lymphatic vessel invasion*				
No	Reference	–	Reference	–
Yes	4.232(1.91–9.374)	0.000	3.294(1.771–9.268)	0.024
*Tumor size*				
≤ 2 cm	Reference	–		
≤ 5 cm	0.259(0.734–6.952)	0.155		
> 5 cm	2.965(0.986–8.917)	0.053		
*Examined LNs*				
≤ 16	Reference	–		
> 16	0.595(0.269–1.317)	0.2

**Table 5:** Basic information of extracted patients from SEER diagnosed in 2010–2015 after propensity–score matching

Variables	Total	Early-onset SRCC	Late-onset SRCC	P value
Total	464	232	232	
*Sex*				0.925
Male	189 (40.73%)	94 (40.52%)	95 (40.95%)	
Female	275 (59.27%)	138 (59.48%)	137 (59.05%)	
Race				0.522
White	310 (66.81%)	156 (67.24%)	154 (66.38%)	
Black	58 (25%)	32 (13.79%)	26 (11.21%)	
Other	96 (20.69%)	44 (18.97%)	52 (22.41%)	
*Primary site*				0.074
Cardia	54 (11.64%)	20 (8.62%)	34 (14.66%)	
Fundus	16 (6.9%)	7 (3.02%)	9 (3.88%)	
Antrum	250 (53.88%)	118 (50.86%)	132 (56.9%)	
Body	66 (14.22%)	39 (16.81%)	27 (11.64%)	
Overlappping/NOS	78 (16.81%)	48 (20.69%)	30 (12.93%)	
*T stage*				0.909
Tis/T1	87 (18.75%)	44 (18.96%)	43 (18.53%)	
T2	64 (13.79%)	30 (12.93%)	34 (14.66%)	
T3	149 (32.11%)	73 (31.47%)	76 (32.76%)	
T4	164 (70.69%)	85 (36.64%)	79 (34.05%)	
*N stage*				0.981
N0	169 (36.42%)	84 (36.21%)	85 (36.64%)	
N1	71 (15.3%)	37 (15.95%)	34 (14.66%)	
N2	90 (19.4%)	44 (18.97%)	46 (19.83%)	
N3	134 (28.88%)	67 (28.88%)	67 (28.88%)	
*M stage*				0.011
M0	399 (86%)	190 (80.34%)	209 (89.66%)	
M1	65 (14%)	42 (19.66%)	23 (10.34%)	
*Tumor number*				1
1	431 (92.89%)	215 (92.68%)	216 (93.1%)	
> 1	33 (7.11%)	17 (7.32%)	16 (6.9%)	
*Tumor size*				0.926
≤ 2 cm	102 (21.98%)	50 (21.55%)	52 (22.41%)	
≤ 5 cm	174 (37.5%)	89 (38.36%)	85 (36.64%)	
> 5 cm	188 (40.52%)	93 (40.09%)	95 (40.95%)	
*Examined LNs*				0.641
≤ 16	213 (45.91%)	104 (44.87%)	109 (49.06%)	
> 16	251 (54.09%)	128 (55.13%)	123 (50.94%)	
*Chemotherapy*				0.849
No	182 (39.22%)	90 (38.79%)	92 (39.66%)	
Yes	282 (60.78%)	142 (61.21%)	140 (60.34%)

**Table 6:** Basic information of included patients from our hospital diagnosed in 2003–2019 after PSM

Variables	Total	Early-onset GSRCC	Late-onset GSRCC	P value
Total	70	35	35	
*Sex*				1
Male	40 (57.14%)	20 (57.14%)	20 (57.14%)	
Female	30 (42.86%)	15 (42.86%)	15 (42.86%)	
*Smoke*				0.255
Never	54 (77.14%)	29 (82.86%)	25 (71.43%)	
Yes	16 (22.86%)	6 (17.14%)	10 (28.57%)	
*Primary site*				0.4
Cardia	3 (4.29%)	1 (2.85%)	2 (5.71%)	
Fundus	3 (4.29%)	1 (2.85%)	2 (5.71%)	
Antrum	42 (60%)	19 (54.28%)	23 (65.71%)	
Body	20 (28.57%)	13 (37.14%)	7 (20%)	
Overlappping/NOS	2 (2.86%)	1 (2.85%)	1 (2.85%)	
*T stage*				0.576
T1	8 (11.43%)	4 (11.43%)	4 (11.43%)	
T2	10 (14.28%)	5 (14.29%)	5 (14.29%)	
T3	10 (14.28%)	7 (20%)	3 (8.57%)	
T4	42 (60%)	19 (54.28%)	23 (65.71%)	
*N stage*				0.63
N0	139 (34.49%)	9 (25.71%)	8 (22.86%)	
N1	60 (14.89%)	2 (5.71%)	5 (14.29%)	
N2	19 (27.14%)	9 (25.71%)	10 (28.57%)	
N3	27 (72.86%)	15 (42.86%)	12 (34.29%)	
*M stage*				0.045
M0	59 (84.29%)	26 (74.29%)	33 (94.29%)	
M1	11 (15.71%)	9 (25.71%)	2 (5.71%)	
*Lesion classification*				0.689
Infiltration type	6 (8.57%)	4 (11.43%)	2 (5.71%)	
Ulcerative type	52 (74.29%)	25 (71.43%)	27 (77.14%)	
Protuberant type	12 (17.14%)	6 (17.14%)	6 (17.14%)	
*Lymphatic vessel invasion*				0.621
No	26 (37.14%)	12 (34.29%)	14 (40%)	
Yes	44 (62.86%)	23 (65.71%)	21(60%)	
*Tumor size*				0.203
≤ 2 cm	22 (31.43%)	8 (22.86%)	14 (40%)	
≤ 5 cm	33 (47.14%)	20 (57.14%)	13 (37.14%)	
> 5 cm	15 (21.43%)	7 (20%)	8 (22.86%)	
*Examined LNs*				0.495
≤ 16	10 (14.29%)	6 (17.14%)	4 (11.43%)	
> 16	60 (85.71%)	29 (82.86%)	31 (88.57%)	
*Chemotherapy*				1
No	38 (54.29%)	19 (54.29%)	19 (54.29%)	
Yes	32 (45.71%)	16 (45.71%)	16 (45.71%)	

## Discussion

SRCC is a kind of rare GC and predicts poor survival. Although the total incidence of GC has decreased, that of SRCC is on the rise [11]. In general, SRCC is known to have a female predominance, to comprise a younger population and to be located in the middle and distal stomach [12]. According to data from American, SRCC is more common in black people [11, 12]. A large proportion of patients are at a late stage when diagnosed and even have distant metastasis, resulting from the insidious symptoms and high malignancy of SRCC [12]. Moreover, approximately 10% of GC is detected in those younger than 45 years old, a trend that is increasing [13]. However, as for early-onset SRCC, some have reported a knowledge gap [14, 15]. In our study, we extracted 2052 cases from the SEER database and 403 from our hospital. Univariate and multivariate analyses revealed that age at 45 years or younger was an independent risk factor for metastasis, as demonstrated by PSM with SEER data and our data. To our knowledge, this is the first study to illustrate the disparity of metastasis between early-onset and late-onset SRCC by the SEER database and validate it in an external group.

EOGC has increased over the past several decades and is highlighted as a challenging compared to LOGC. With regard to genetic variation, TP53 mutation occurs less often in EOGC than in LOGC, whereas MUC5B, CDH1, and TGFBR1 present higher mutation rates, demonstrating that the poor prognosis for EOGC might be associated with these mutated genes [16]. The histopathological characteristics and clinical behaviour are also quite distinct between EOGC and LOGC. For example, early-onset colorectal cancer is reported to more frequently present with lymph node metastasis or distant metastasis [17, 18]. Another study using the SEER database found that the proportion of EOGC patients with metastatic disease was greater than that of LOGC patients (49.5 vs 40.9%, P < 0.01) [15]. In line with this previous study [19], our results showed that early-onset SRCC patients from the SEER database more commonly had metastasis (19.66% vs 10.34%, P < 0.001), and early-onset SRCC patients from our hospital had similar clinical characteristics (20.37% vs 9.17%, P < 0.05). Furthermore, multivariate logistic regression analysis demonstrated that age at 45 years or younger was a risk factor, which was consistent with the results of the PSM analysis. Multivariate analysis and PSM analysis can avoid the effect of confounding factors, making our results reliable. There are some potential explanations from different aspects for these findings. Regarding genomic mutations, several studies have reported that early-onset SRCC is associated with a de novo deletion of CDH1, which encodes a protein functioning as an adherens junction; this to some extent explains why early-onset SRCC is more likely to be metastatic [15, 20]. Regarding clinical characteristics, the distinct sex distribution between early-onset SRCC and late-onset SRCC may also account for the high malignancy [21]. In addition to distant metastasis, we found that lymphatic vessel invasion was more frequent in early-onset patients, which suggests that early-onset SRCC has greater malignancy.

In line with other studies [15, 22], we found that early-onset SRCC patients had a better prognosis than late-onset patients base on SEER data. Although the results could not be validated by data from our hospital because of the limited number of patients, we provide some explanations. We found that the proportion of those who underwent chemotherapy was larger in early-onset patients than in late-onset patients (Table 2), which somewhat explains the result. Similarly, other studies found that chemoradiotherapy was more frequent in early-onset patients; moreover, the complication of surgery for early-onset patients was less than that for late-onset patients [23]. In early-onset patients, the main cause of death is advanced disease, and in older patients, it is due to associated comorbid conditions. In addition, mutation of TP53 occurs less often in EOGC than in LOGC, whereas MUC5B, CDH1, and TGFBR1 present higher mutation rates, demonstrating that the poor prognosis of EOGC might be associated with these mutated genes [16]. Overall, survival between early-onset and late-onset GC remains controversial, and some studies report that when matched for imbalanced information, the prognosis does not differ from that of late-onset patients [24].

Our study has some limitations that should be discussed. First, TNM staging in the SEER database was performed using the 7^th^ edition rather than the 8th edition, which may influence the identification of risk factors for metastasis. However, our own data assessed TNM staging according to the 8th edition, which compensated for this defect. Similarly, some important factors, such as lymphatic invasion and smoking, are not recorded in the SEER database, but these data were available at our hospital. Then, we excluded many patients who had missing data associated with our collected variables, increasing selection bias. Next, variables including examined LNs and positive LNs depended on each doctor in different clinical centres. Finally, we only enrolled patients who received surgical resection, which can be a critical limitation and affect our results to some degree. Our study has some advantages, such as internal and external validation and complementary roles between the two sets of data.

## Conclusion

In conclusion, our study showed that distant metastasis is more common in early-onset SRCC than in late-onset SRCC. The increased frequency of distant metastasis in SRCC patients younger than 45 years of age offers a unique opportunity to gain a better understanding of carcinogenesis, which might be exploited during diagnosis and management. Regardless, further studies are needed to explore the potential aetiologic basis for the disparity.

## Data Availability

The datasets generated and/or analysed during the current study are available in the SEER repository (https://seer.cancer.gov/data/).

## Abbreviations

SEER: Surveillance, epidemiology, and end results
SRC: Signet ring cell carcinoma
GC: Gastric cancer
EOGC: Early-onset GC
LOGC: Late-onset GC
